# Chatting Up the Next Big Thing: Generating Novel Ideas for Systematic Reviews in Breast Reconstruction Research Using Chat-GPT

**DOI:** 10.7759/cureus.61955

**Published:** 2024-06-08

**Authors:** Souha Farhat, Alexa De la Fuente Hagopian, Karan Hooda, Hung B Le, Anthony Echo

**Affiliations:** 1 The Institute for Reconstructive Surgery, Houston Methodist Hospital, Weill Cornell Medicine, Houston, USA; 2 Department of Plastic and Reconstructive Surgery, Engineering Medicine (EnMed) Program, Texas A&M Health Science Center, Houston, USA; 3 Department of Plastic and Reconstructive Surgery, Dell Medical School, Austin, USA

**Keywords:** breast reconstruction, reconstructive plastic surgery, plastic and reconstructive surgery, systematic review, research, novel ideas, chat-gpt, reconstructive breast surgery

## Abstract

Background: In reconstructive plastic surgery, the need for comprehensive research and systematic reviews is apparent due to the field’s intricacies, influencing the evidence supporting specific procedures. Although Chat-GPT's knowledge is limited to September 2021, its integration into research proves valuable for efficiently identifying knowledge gaps. Therefore, this tool becomes a potent asset, directing researchers to focus on conducting systematic reviews where they are most necessary.

Methods: Chat-GPT 3.5 was prompted to generate 10 unpublished, innovative research topics on breast reconstruction surgery, followed by 10 additional subtopics. Results were filtered for systematic reviews in PubMed, and novel ideas were identified. To evaluate Chat-GPT’s power in generating improved responses, two additional searches were conducted using search terms generated by Chat-GPT.

Results: Chat-GPT produced 83 novel ideas, leading to an accuracy rate of 83%. There was a wide range of novel ideas produced among topics such as transgender women, generating 10 ideas, whereas acellular dermal matrix (ADM) generated five ideas. Chat-GPT increased the total number of manuscripts generated by a factor of 2.3, 3.9, and 4.0 in the first, second, and third trials, respectively. While the search results were accurate to our manual searches (95.2% accuracy), the greater number of manuscripts potentially diluted the quality of articles, resulting in fewer novel systematic review ideas.

Conclusion: Chat-GPT proves valuable in identifying gaps in the literature and offering insights into areas lacking research in breast reconstruction surgery. While it displays high sensitivity, refining its specificity is imperative. Prudent practice involves evaluating accomplished work and conducting a comprehensive review of all components involved.

## Introduction

Chat-GPT is a public tool developed by OpenAI. Its underlying technology, generative pertained transformer (GPT) is a creative, powerful artificial intelligence (AI) design capable of generating answers to inquiries quickly and reasonably using an intelligent algorithm that allows it to converse like a human. Although Chat-GPT's 3.5 extensive knowledge is limited to September 2021, Chat-GPT has exploded in the past year since its release in November 2022 in various fields, such as education, customer service, marketing, gaming, and creative writing [1]. At the time of this manuscript submission, Chat-GPT 4.0 was released, which includes data up until May 2023. In the medical field, Chat-GPT’s potential to revolutionize medicine has been highlighted in an evaluation of its successful performance on the three United States Medical Licensing Examinations. Without any previous training, ChatGPT was able to score a passing grade on all three examinations [2]. There are still some worthwhile avenues through which Chat-GPT can be integrated into medical research to improve the research process. The research performed by academic clinicians is a vital part of medical and surgical advancement. However, the necessary journey from idea to publication can take a long time. Reviewing the peer-reviewed literature is crucial in ensuring that the idea of interest is innovative and valuable as it puts articles to greater scrutiny by other experts in the field, producing higher-quality research results and advancing the field [3]. In the hierarchy of evidence, systematic reviews and meta-analyses represent the highest level of evidence among different types of research studies. By synthesizing the findings of multiple studies, especially when findings are conflicting, they provide the most comprehensive and reliable foundation for informed decision-making in healthcare and various other fields. Now, with the introduction of AI, Chat-GPT can be integrated into research as a novel avenue for facilitating research by generating innovative research ideas and inquiries, thereby aiding the creation of systematic reviews that can address critical questions posed by healthcare providers [4].

Reconstructive plastic surgery is a discipline of remarkable intricacy and complexity, which seeks to restore form and function to injured, disfigured, or congenitally deformed body parts. Through sophisticated surgical techniques, this field aims to repair, reconstruct, and rejuvenate the human body. With the complexity found in the field, there is also a need for more research and systematic reviews, significantly affecting the evidence for the efficacy of specific procedures. Its implementation into the research of reconstructive plastic surgery, as within any field, can serve as a powerful tool for identifying knowledge gaps efficiently and effectively, enabling researchers to focus on conducting and publishing systematic reviews where they are most needed.

Herein, we aim to achieve two objectives: to assess the efficiency and precision of Chat-GPT 3.5 as an aid in the generation of innovative ideas for systematic reviews within the field of breast reconstruction research and to investigate Chat-GPT's ability to formulate search terms that can enhance and streamline the systematic review search process.

## Materials and methods

Purpose 1: Evaluating Chat-GPT’s use in generating novel ideas for systematic reviews in breast reconstruction research

**Figure 1:**
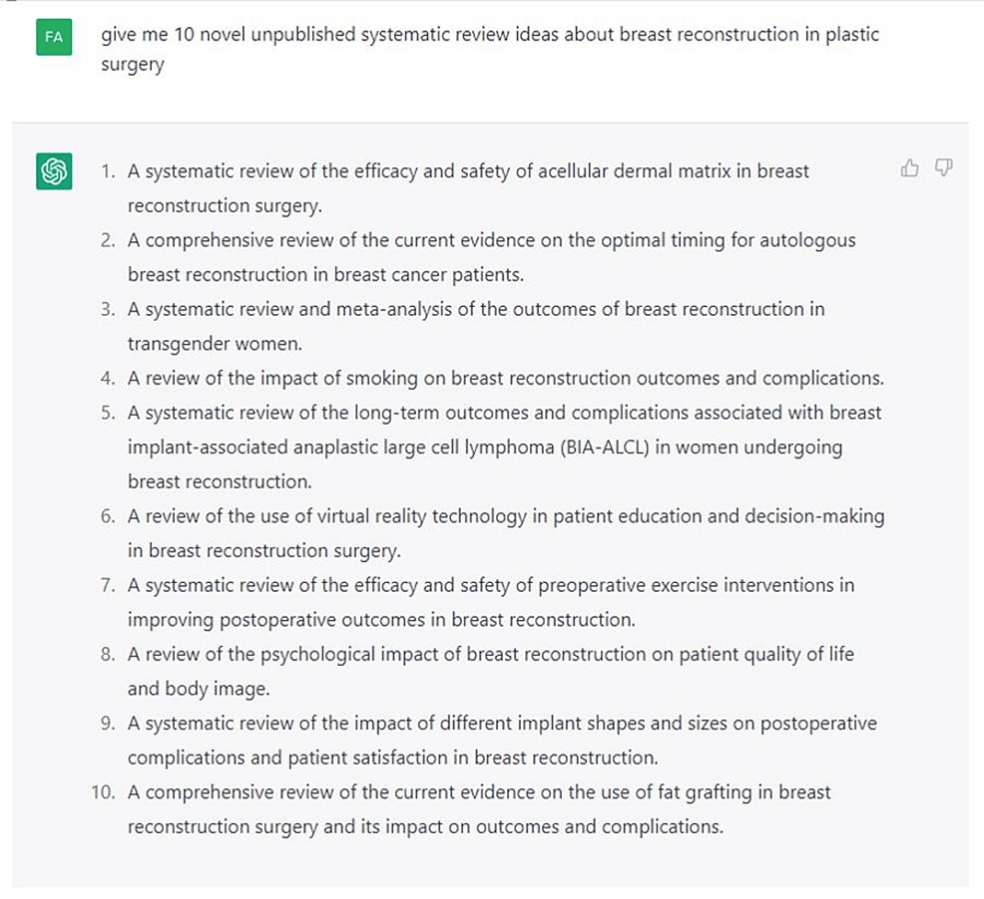
Inquiry that led to the generation of the 10 unpublished systematic review ideas using Chat-GPT.

**Figure 2:**
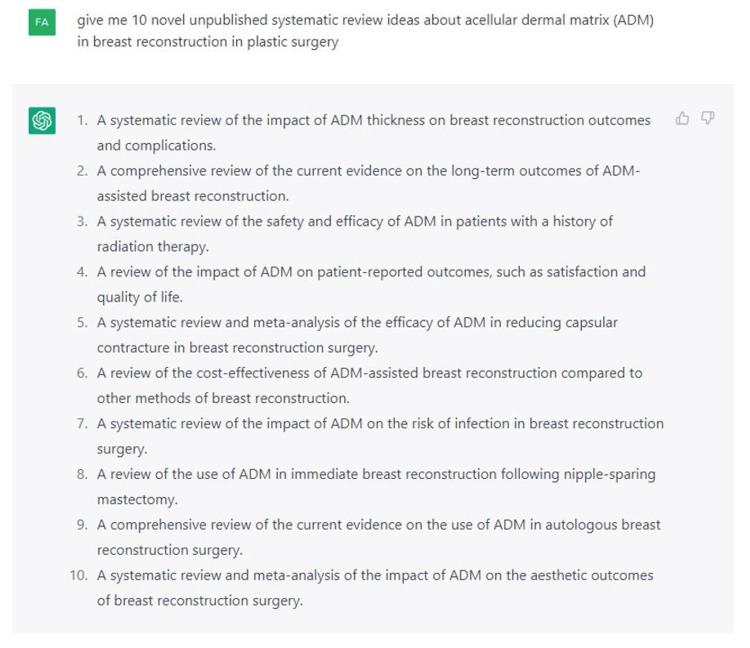
Inquiry that led to the generation of a list of 10 subtopics related to the acellular dermal matrix using Chat-GPT.

We first requested Chat-GPT 3.5 to generate 10 unpublished, innovative research topics followed by 10 additional subtopics related to breast reconstructive surgery. From there, Chat-GPT was prompted to “create a table with 10 unpublished, novel research topics on breast reconstructive surgery (Figure 1), followed by 10 additional subtopics within each topic (example of acellular dermal matrix in Figure 2).

**Figure 3:**
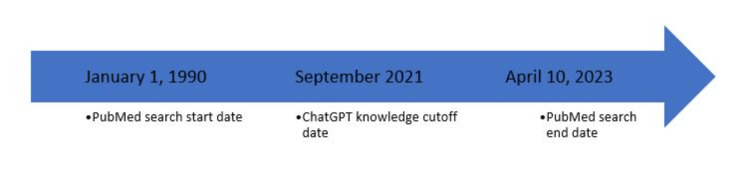
Timeline showing the dates pertinent to the search performed.

Once we finalized the initial result from Chat-GPT, the PubMed database was searched using our search terms based on the 100 total topics generated by Chat-GPT. Then, a filter for systematic reviews was implemented on the PubMed database spanning the period from January 1, 1990, to April 10, 2023. Non-English manuscripts were excluded from the search. The timeline of the dates pertinent to the search performed is represented in Figure 3.

In cases where no systematic review manuscript was found, the idea was counted as novel and documented as such. This step was repeated for all 10 subtopics. All data were imported into Excel (Microsoft Corporation, Redmond, WA), which was employed to conduct statistical analysis and create the relevant tables. ChatGPT was not utilized to write any part of this manuscript.

Purpose 2: Exploring Chat-GPT's ability to formulate search terms that can improve the systematic review search process

**Figure 4:**
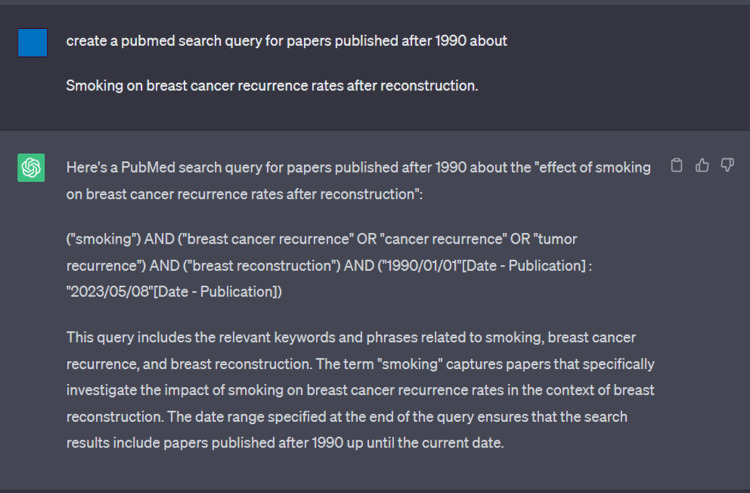
Trial 1 of MESH created from Chat-GPT for search queries through PubMed. For this specific query, Trials 2 and 3 yielded the same result.

Chat-GPT was used to generate search terms for every topic among the 10 generated by Chat-GPT (Figure 4). To evaluate Chat-GPT’s power in generating improved responses when prompted to do so, two additional searches were conducted using search terms generated by Chat-GPT. This was done by using the “regenerate” option within Chat-GPT. The process found in Figures 1-2 described in Purpose 1 of the Methods section was repeated.

Once all of the data were collected for Purpose #1 and #2, we did a qualitative analysis of the data using standard deviation and mean to compare the novelty created by Chat-GPT for both purposes.

## Results

Purpose 1: Evaluating Chat-GPT’s use in generating novel ideas for systematic reviews in breast reconstruction research

Chat-GPT generated 100 systematic review subtopics within 10 topics related to the field of breast reconstruction in plastic surgery. After browsing the literature using PubMed, we found that Chat-GPT was able to produce 83 novel ideas for systematic reviews (Table 1), leading to an 83% accuracy rate.

**Table 1:** Total number of manuscripts, systematic reviews, and novel systematic reviews based on our PubMed search.

Topics	Total Number of Manuscripts	Total Number of Systematic Reviews	Number of Novel Systematic Reviews Ideas (%)
Acellular Dermal Matrix	457	17	5 (50)
Autologous Breast Reconstruction	438	16	5 (50)
Transgender Women Transition	9	0	10 (100)
Smoking	229	5	7 (70)
BIA-ALCL	2	0	10 (100)
Virtual Reality	1	0	10 (100)
Pre-operative Exercise Intervention	5	0	10 (100)
Psychological Impact	33	0	10 (100)
Implant Shapes and Sizes	63	0	10 (100)
Fat Grafting	89	10	6 (60)
Total	1326	48	83 (83)

The ADM topic had the highest number of systematic reviews published with 17, followed by autologous breast reconstruction with 16, fat grafting with 10, and smoking with five. The other topics such as transgender women's transition, breast implant-associated anaplastic large cell lymphoma (BIA-ALCL), and virtual reality did not have any published systematic reviews. All the topics generated by Chat-GPT had non-systematic review papers published.

Purpose 2: Exploring Chat-GPT's ability to formulate search terms that can improve the systematic review search process

In Trial 1 of Chat-GPT’s search, 81 systematic reviews were found to be novel compared to 83 found when we searched PubMed using our search terms. This corresponds to an accuracy of 97.6%. The breakdown of the results of Chat-GPT’s first trial of search terms for 10 topics with their respective accuracy rates in relation to our search is represented in Table 2. The subtopics and the novelty of systematic reviews published based on Chat-GPT’s first trial are shown in Tables 3-4.

**Table 2:** Comparison of novel ideas generated by our search vs Chat-GPT and accuracy rate based on Trial 1.

Topics	Our Search Results			Trial 1 Chat-GPT Results			Accuracy (%)
	Total Number of Manuscripts	Total Number of Systematic Reviews	Number of Novel Systematic Reviews	Total Number of Manuscripts	Total Number of Systematic Reviews	Number of Novel Systematic Reviews	
Acellular Dermal Matrix	457	17		865	36	2	40
Autologous Breast Reconstruction	438	16	5	139	3	9	20
Transgender Women Transition	9	0	10	0	0	10	100
Smoking	229	5	7	1608	29	5	71.4
BIA-ALCL	2	0	10	724	20	7	70
Virtual Reality	1	0	10	4	0	10	100
Pre-operative Exercise Intervention	5	0	10	0	0	10	100
Psychological Impact	33	0	10	773	21	8	80
Implant Shapes and Sizes	63	0	10	8	0	10	100
Fat Grafting	89	10	6	3	0	10	33.3
Total			83			81	97.6

**Table 3:** Total number of ideas for systematic reviews for two topics generated by Chat-GPT and their novelty based on Chat-GPT’s generated search terms (Trial 1). See Table 4 for the other eight topics.

Topic	Subtopics	# Systematic Reviews N: novel	# Novel Systematic Review Ideas
Acellular Dermal Matrix	A systematic review of the impact of ADM thickness on breast reconstruction outcomes and complications.	N	2
	A comprehensive review of the current evidence on the long-term outcomes of ADM-assisted breast reconstruction.	1	
	A systematic review of the safety and efficacy of ADM in patients with a history of radiation therapy.	1	
	A review of the impact of ADM on patient-reported outcomes, such as satisfaction and quality of life.	7	
	A systematic review and meta-analysis of the efficacy of ADM in reducing capsular contracture in breast reconstruction surgery.	6	
	A review of the cost-effectiveness of ADM-assisted breast reconstruction compared to other methods of reconstruction.	1	
	A systematic review of the impact of ADM on the risk of infection in breast reconstruction surgery.	14	
	A review of the use of ADM in immediate breast reconstruction following nipple-sparing mastectomy.	1	
	A comprehensive review of the current evidence on the use of ADM in autologous breast reconstruction surgery.	N	
	A systematic review and meta-analysis of the impact of ADM on the aesthetic outcomes of breast reconstruction surgery.	5	
Transgender Women Transition	A systematic review and meta-analysis of the long-term outcomes of breast reconstruction surgery in transgender women, including patient-reported satisfaction and quality of life.	N	10
	A review of the impact of hormone therapy on breast reconstruction outcomes in transgender women.	N	
	A systematic review of the safety and efficacy of different breast reconstruction techniques in transgender women.	N	
	A review of the psychological impact of breast reconstruction surgery on body image and self-esteem in transgender women.	N	
	A comprehensive review of the current evidence on the optimal timing for breast reconstruction surgery in transgender women.	N	
	A systematic review of the impact of race and ethnicity on breast reconstruction outcomes in transgender women.	N	
	A review of the impact of smoking and other lifestyle factors on breast reconstruction outcomes in transgender women.	N	
	A systematic review of the impact of insurance coverage on access to and outcomes of breast reconstruction surgery in transgender women.	N	
	A review of the use of autologous tissue transfer in breast reconstruction surgery for transgender women and its impact on outcomes and complications.	N	
	A systematic review of the impact of breast size and shape preferences on breast reconstruction outcomes and patient satisfaction in transgender women.	N	

**Table 4:** Total number of ideas for systematic reviews for the eight remaining topics generated by Chat-GPT and their novelty based on Chat-GPT’s generated search terms (continued).

Topic	Subtopic	# Systematic Reviews N: novel	# Novel Systematic Review Ideas
Autologous Breast Reconstruction	Comparison of different surgical techniques for autologous breast reconstruction.	3	9
	Comparison of outcomes and complications of autologous breast reconstruction vs. implant-based reconstruction.	N	
	Evaluation of the impact of smoking and other modifiable risk factors on the outcomes of autologous breast reconstruction.	N	
	Analysis of the effect of age on the outcomes of autologous breast reconstruction.	N	
	Assessment of the impact of adjuvant therapy (chemotherapy, radiation therapy) on the outcomes of autologous breast reconstruction.	N	
	Investigation of the role of preoperative breast imaging in the planning and success of autologous breast reconstruction.	N	
	Evaluation of the long-term outcomes of autologous breast reconstruction, including aesthetic results and patient satisfaction.	N	
	Comparison of the cost-effectiveness of autologous breast reconstruction vs. implant-based reconstruction.	N	
	Analysis of the impact of body mass index (BMI) on the outcomes of autologous breast reconstruction.	N	
	Assessment of the effect of comorbidities (e.g., diabetes, hypertension) on the outcomes of autologous breast reconstruction.	N	
Smoking	Evaluation of the impact of smoking on the outcomes of breast reconstruction, including complications and patient satisfaction.	9	5
	Comparison of outcomes and complications of breast reconstruction in smokers vs. non-smokers.	9	
	Assessment of the impact of smoking cessation on the outcomes of breast reconstruction.	N	
	Investigation of the relationship between smoking and wound healing complications in breast reconstruction.	8	
	Evaluation of the impact of smoking on the success of autologous breast reconstruction vs. implant-based reconstruction.	2	
	Analysis of the effect of smoking on the outcomes of breast reconstruction in patients with comorbidities such as diabetes, hypertension, or obesity.	N	
	Comparison of the cost-effectiveness of breast reconstruction in smokers vs. non-smokers.	N	
	Assessment of the effect of secondhand smoke exposure on the outcomes of breast reconstruction.	N	
	Evaluation of the impact of smoking on the use of acellular dermal matrix in breast reconstruction.	1	
	Investigation of the impact of smoking on breast cancer recurrence rates after reconstruction.	N	
BIA-ALCL	A systematic review and meta-analysis of the incidence and risk factors of BIA-ALCL in women undergoing breast reconstruction with implants.	N	7
	A comprehensive review of the current evidence on the optimal diagnostic and treatment strategies for BIA-ALCL in women undergoing breast reconstruction with implants.	N	
	A systematic review of the impact of implant characteristics, such as type, size, and surface texture, on the risk of developing BIA-ALCL in women undergoing breast reconstruction.	N	
	A review of the impact of patient factors, such as age, comorbidities, and genetic predisposition, on the risk of developing BIA-ALCL in women undergoing breast reconstruction with implants.	2	
	A systematic review of the safety and efficacy of different surgical techniques for implant removal and treatment of breast implant-associated anaplastic large cell lymphoma in women undergoing breast reconstruction.	N	
	A review of the psychological impact of breast implant-associated anaplastic large cell lymphoma on patient satisfaction and quality of life in women undergoing breast reconstruction with implants.	17	
	A comprehensive review of the current evidence on the long-term outcomes and prognosis of breast implant-associated anaplastic large cell lymphoma in women undergoing breast reconstruction with implants.	1	
	A systematic review of the impact of different implant monitoring strategies, such as imaging and seroma testing, on the detection and management of breast implant-associated anaplastic large cell lymphoma in women undergoing breast reconstruction with implants.	N	
	A review of the impact of patient education and informed consent on the incidence and management of breast implant-associated anaplastic large cell lymphoma in women undergoing breast reconstruction with implants.	N	
	A systematic review and meta-analysis of the effectiveness of different regulatory and surveillance policies on the prevention and management of breast implant-associated anaplastic large cell lymphoma in women undergoing breast reconstruction with implants.	N	
Virtual Reality	A systematic review and meta-analysis of the impact of virtual reality technology on patient outcomes and satisfaction in breast reconstruction surgery.	N	10
	A comprehensive review of the current evidence on the use of virtual reality technology in preoperative planning and surgical simulation for breast reconstruction.	N	
	A systematic review of the impact of virtual reality technology on the accuracy and precision of breast reconstruction surgery.	N	
	A review of the impact of virtual reality technology on the learning curve and training of surgeons performing breast reconstruction surgery.	N	
	A systematic review of the impact of virtual reality technology on patient education and informed consent in breast reconstruction surgery.	N	
	A review of the impact of virtual reality technology on the psychological well-being and quality of life of patients undergoing breast reconstruction surgery.	N	
	A comprehensive review of the current evidence on the feasibility and cost-effectiveness of implementing virtual reality technology in breast reconstruction surgery.	N	
	A systematic review of the impact of virtual reality technology on intraoperative decision-making and surgical outcomes in breast reconstruction.	N	
	A review of the impact of virtual reality technology on patient-reported pain, discomfort, and recovery time in breast reconstruction surgery.	N	
	A systematic review of the impact of virtual reality technology on postoperative monitoring and follow-up care for patients undergoing breast reconstruction.	N	
Pre-operative Exercise Intervention	Evaluation of the impact of pre-operative exercise on post-operative pain in breast reconstruction.	N	
	Comparison of outcomes and complications of breast reconstruction in patients who underwent pre-operative exercise intervention.	N	10
	Assessment of the impact of pre-operative exercise on the duration of hospitalization in breast reconstruction.	N	
	Investigation of the relationship between pre-operative exercise and post-operative functional outcomes in breast reconstruction.	N	
	Evaluation of the impact of pre-operative exercise on the incidence of post-operative lymphedema in breast reconstruction.	N	
	Analysis of the effect of pre-operative exercise on patient-reported outcomes in breast reconstruction, such as quality of life and satisfaction.	N	
	Comparison of the cost-effectiveness of breast reconstruction in patients who underwent pre-operative exercise intervention.	N	
	Assessment of the effect of the timing and duration of pre-operative exercise intervention on post-operative outcomes in breast reconstruction.	N	
	Evaluation of the impact of pre-operative exercise on post-operative infection rates in breast reconstruction.	N	
	Investigation of the impact of pre-operative exercise on the use of opioids in breast reconstruction.	N	
Psychological Impact	A systematic review and meta-analysis of the impact of breast reconstruction on body image and self-esteem in breast cancer survivors.	19	8
	A comprehensive review of the current evidence on the impact of breast reconstruction on sexual function and intimacy in breast cancer survivors.	N	
	A systematic review of the impact of breast reconstruction on social and occupational functioning in breast cancer survivors.	N	
	A review of the psychological impact of different types of breast reconstruction procedures on patient satisfaction and quality of life.	N	
	A systematic review of the impact of patient characteristics, such as age, race, and socioeconomic status, on the psychological outcomes of breast reconstruction.	N	
	A review of the impact of timing and delay of breast reconstruction on the psychological well-being of breast cancer survivors.	N	
	A comprehensive review of the current evidence on the impact of breast reconstruction on post-traumatic growth and resilience in breast cancer survivors.	N	
	A systematic review of the impact of breast reconstruction on anxiety, depression, and other psychological symptoms in breast cancer survivors.	2	
	A review of the impact of patient education and informed consent on the psychological outcomes of breast reconstruction.	N	
	A systematic review of the impact of psychotherapeutic interventions, such as cognitive-behavioral therapy and support groups, on the psychological well-being of breast cancer survivors undergoing breast reconstruction.	N	
Implant Shapes and Sizes	A systematic review and meta-analysis of the impact of implant size on patient satisfaction and quality of life after breast augmentation surgery.	N	10
	A comprehensive review of the current evidence on the impact of implant shape on patient-reported outcomes and complications after breast augmentation surgery.	N	
	A systematic review of the impact of implant size and shape on the aesthetic outcomes of breast augmentation surgery.	N	
	A review of the impact of implant size and shape on the risk of complications, such as capsular contracture and implant malposition, after breast augmentation surgery.	N	
	A systematic review of the impact of implant size and shape on the incidence of implant rupture and need for revision surgery after breast augmentation.	N	
	A review of the impact of implant size and shape on the psychological well-being and quality of life of patients undergoing breast augmentation surgery.	N	
	A comprehensive review of the current evidence on the impact of implant size and shape on the long-term outcomes and durability of breast augmentation surgery.	N	
	A systematic review of the impact of implant size and shape on patient-reported pain, discomfort, and recovery time after breast augmentation surgery.	N	
	A review of the impact of patient education and informed consent on the decision-making process regarding implant size and shape in breast augmentation surgery.	N	
	A systematic review and meta-analysis of the impact of different implant sizing systems and techniques on the accuracy and precision of implant selection in breast augmentation surgery.	N	
Fat Grafting	A systematic review and meta-analysis of the efficacy of fat grafting in breast reconstruction surgery and its impact on aesthetic outcomes.	N	10
	A review of the safety and efficacy of different techniques for fat grafting in breast reconstruction surgery.	N	
	A systematic review of the impact of adipose tissue quality on the outcomes and complications of fat grafting in breast reconstruction surgery.	N	
	A comprehensive review of the current evidence on the optimal timing for fat grafting in breast reconstruction surgery.	N	
	A review of the psychological impact of fat grafting in breast reconstruction surgery on patient satisfaction and quality of life.	N	
	A systematic review of the impact of radiation therapy on fat grafting outcomes in breast reconstruction surgery.	N	
	A review of the impact of obesity and weight fluctuations on fat grafting outcomes in breast reconstruction surgery.	N	
	A systematic review of the safety and efficacy of fat grafting in immediate breast reconstruction following nipple-sparing mastectomy.	N	
	A review of the impact of patient factors, such as age and comorbidities, on fat grafting outcomes and complications in breast reconstruction surgery.	N	
	A comprehensive review of the current evidence on the use of fat grafting in combination with other breast reconstruction techniques and its impact on outcomes and complications.	N	

The first, second, and third search strategies suggested by Chat GPT yielded 3.1, 4.7, and 5.6 times more results for the total number of manuscripts than our search, respectively. Similarly, the first, second, and third search strategies yielded 2.3, 3.9, and 4.0 times more results for systematic reviews than our search, respectively. Chat-GPT was able to generate 81 novel ideas in the first two trials and 77 for the third trial with accuracies corresponding to 97.6% and 92.8%, respectively (Table 5).

**Table 5:** Compiled PubMed literature search results for all breast reconstruction topics of our PubMed search; Trials 1, 2, and 3 of Chat-GPT-created MESH terms for PubMed data, detailing the accuracy of Chat-GPT.

		Total # of Manuscripts	Results yield factor of Chat-GPT search compared to ours for Non-Systematic Reviews	# of Systematic Reviews	Results yield factor of Chat-GPT search compared to ours for Systematic Reviews	# of Non-novel Ideas	# of Novel Systematic Reviews	Accuracy Rate (%)
Our PubMed Search		1278		48		17	83	
Chat-GPT Trial #	1	4015	3.1	109	2.3	19	81	97.6
	2	6052	4.7	187	3.9	19	81	97.6
	3	7151	5.6	194	4.0	23	77	92.8

## Discussion

ChatGPT's ability to generate novel research ideas in breast reconstruction surgery is evidenced by its rapid creation of 83 unique systematic reviews. However, disparities persist within this research domain, highlighted by limited growth in specific subtopics. Notably, a paucity of systematic reviews exists for breast reconstruction in transgender women, with Chat-GPT producing 10 novel ideas within this field. In contrast, ADM, and autologous breast reconstruction, for which Chat-GPT has generated five novel systematic review ideas, is accompanied by a surplus of existing literature. Therefore, Chat-GPT’s ability to generate novel ideas for systematic reviews allows researchers to detect gaps in the literature and provides valuable insight into the areas where research is lacking in the field of breast reconstruction surgery.

ChatGPT’s power was also tapped into when prompted to suggest MESH terms and keywords when generating a search strategy on topics related to breast reconstruction. When prompted to regenerate other answers, Chat-GPT increased the total number of manuscripts generated by a factor of 3.1, 4.7, and 5.6 in the first, second, and third trials, respectively. Similarly, Chat-GPT increased the total number of systematic reviews generated by a factor of 2.3, 3.9, and 4.0 in the first, second, and third trials, respectively. This indicates that, while ChatGPT excels at extracting a substantial volume of literature by generating unique MESH terms, its ability to provide specific insights is somewhat constrained. This trend can be observed when comparing our own search data to Chat-GPT in Table 2, particularly in Trial 1. Although Chat-GPT's search results were accurate to our own, many of the topics had a greater number of manuscripts obtained, thus potentially diluting the quality of the articles, resulting in less novel systematic review ideas. Therefore, while Chat-GPT displays a high level of sensitivity in facilitating systematic reviews, its specificity requires further refinement. Thus, prudent practice involves not only evaluating the work accomplished but also conducting a comprehensive review of all components involved.

Since its release, Chat-GPT’s abilities have been tested and incorporated in various fields, including plastic surgery. This is demonstrated in the increasing number of publications in the last few months addressing Chat-GPT’s potential role in various aspects of plastic surgery. As of December 21, 2023, over 70 articles concerning ChatGPT in plastic surgery have been published, indicating a growing interest in investigating this technology in this highly innovative field. ChatGPT’s potential is explored not only in enhancing the research process [5-7], writing complete systematic reviews [8], and grants [9] but also in its performance on plastic surgery in-service examination [10]. It has also been found to serve as an aid as a tool for writing patient letters in clinics [2,11]. In this study, Chat-GPT was also found to be fairly accurate in creating novel systematic review ideas specifically in the field of breast reconstruction, with eight of the 10 topics having percent accuracies of 70 or greater (Table 2). Albeit minimal, the change in accuracy percentage between the search results yielded upon using the “regenerate” option provided by Chat-GPT more than once, although yielding a different version of the answer in the first trial, was not found to be correlated with an increased accuracy rate (97.6% for Trials 1 and 2 and 92.8% for Trial 3; Table 5). Hence, using the “regenerate” option may not be necessary for conducting a more thorough search of the literature.

While this paper highlights the potential of Chat-GPT in assisting plastic surgery researchers with literature searches and idea generation, limitations must be acknowledged. The PubMed search employed in this study encompassed publications from January 1, 1990, to April 30, 2023, whereas Chat-GPT's training data cutoff date stands in September 2021. This discrepancy could potentially misrepresent the full extent of Chat-GPT's capabilities. While the study effectively demonstrates its ability to identify relevant research within the specified timeframe, potentially novel ideas published after September 2021 and before April 2023 might not be adequately captured by Chat-GPT but accurately captured by our team’s manual PubMed search. Future research could explore employing alternative knowledge sources alongside Chat-GPT or investigate methods for incorporating more recent publications into its output to address this limitation and provide a more comprehensive evaluation of its potential in plastic surgery research [7]. Additionally, with OpenAI releasing an updated version of Chat-GPT on November 21, 2023 [12], the improved access to medical literature will contribute to its ability to generate increasingly accurate search results and holds significant promise for accelerating research in plastic surgery through advancements in data analysis and communication. However, while the November update undoubtedly holds tremendous potential for research advancement, addressing data accuracy and ethical concerns remains crucial for maximizing its impact. The lack of transparency in Chat-GPT's neural algorithm in its decision-making process and data collection leaves unseen bias that makes analytical research hard to reproduce or verify. An example of ethical concerns around the use of Chat-GPT in writing scientific articles is addressed in a paper published in March 2023 by Najfali et al. where the authors highlight the importance of practicing caution with authorship [13,14].

Limitations

Although Chat-GPT 3.5 rapidly generated novel ideas for systematic reviews, it is constrained by a knowledge cutoff till September 2021, hindering post-2021 insights. Relying solely on crowd-sourced data poses a risk of inaccuracies due to user-contributed nonsensical information. This limitation may limit a comprehensive understanding of the applicability of systematic reviews to real-world challenges in breast reconstruction. Thus, cross-referencing with reputable sources becomes vital to ensure the novelty and relevance of research ideas. Furthermore, certain subtopics in breast reconstruction surgery have garnered substantial attention. Hence, the presence of unexplored facets underscores untapped potential in bridging the knowledge gap in the breast reconstruction literature.

## Conclusions

Leveraging Chat-GPT offers promising avenues to enhance the systematic review process. This technology can aid in developing innovative scoping tools that contribute to more useful systematic reviews. However, it is important to recognize some limitations of Chat-GPT. While it excels at extracting a substantial volume of literature by generating unique MESH terms, its ability to provide specific insights is somewhat constrained. While Chat-GPT displays a high level of sensitivity in facilitating systematic reviews, its specificity requires further refinement. Thus, prudent practice involves not only evaluating the work accomplished but also conducting a comprehensive review of all components involved.
